# A Survey of Universal Quantum von Neumann Architecture

**DOI:** 10.3390/e25081187

**Published:** 2023-08-09

**Authors:** Yuan-Ting Liu, Kai Wang, Yuan-Dong Liu, Dong-Sheng Wang

**Affiliations:** 1 CAS Key Laboratory of Theoretical Physics, Institute of Theoretical Physics, Chinese Academy of Sciences, Beijing 100190, China; 2 School of Physical Sciences, University of Chinese Academy of Sciences, Beijing 100049, China

**Keywords:** quantum computing, von Neumann architecture, quantum entanglement

## Abstract

The existence of universal quantum computers has been theoretically well established. However, building up a real quantum computer system not only relies on the theory of universality, but also needs methods to satisfy requirements on other features, such as programmability, modularity, scalability, etc. To this end, here we study the recently proposed model of quantum von Neumann architecture by putting it in a practical and broader setting, namely, the hierarchical design of a computer system. We analyze the structures of quantum CPU and quantum control units and draw their connections with computational advantages. We also point out that a recent demonstration of our model would require less than 20 qubits.

## 1. Introduction

At the origin of quantum computing, physicists such as R. Feynman and D. Deustch realized that universal quantum computing is possible [1]. It is important to note that, at that time, classical computers were only just being built. After decades of evolution, classical computers have become more and more advanced. In the meantime, the field of quantum information science grows, and nowadays physicists and engineers can control quantum processors of tens or even hundreds of qubits.

As the foundation of computation, physics is not only crucial to guide the finding of elementary devices such as transistors, but also crucial to set the principles of computation regarding space, time, energy, efficiency, etc. However, physics itself is not enough. For the building of classical computers, some other disciplines of study also played essential roles, in particular, the theories of information, systems, and control. Information theory, established by C. Shannon [2], borrows ideas from thermodynamics but it reveals far more properties of information. The system theory, pioneered by von Bertalanffy [3], has more connections with many-body physics and it emphasizes more the structure, correlation, etc., rather than the individual participant. Control theory, from N. Wiener [4], studies the interplay between the controller and the target system to achieve a certain goal. These studies go beyond the traditional scope of physics. Together with computational complexity theory [5], they form the theoretical foundation to make classical computers real.

Modern quantum physics, especially quantum information science, is not a traditional physics; instead, it shares features of engineering. It does not only study systems passively, namely, only studying those that exist naturally, but also actively studies specific systems, e.g., how to make an artificial system for a certain purpose. Therefore, it needs both physicists and engineers to make quantum computers real.

**Figure 1:**
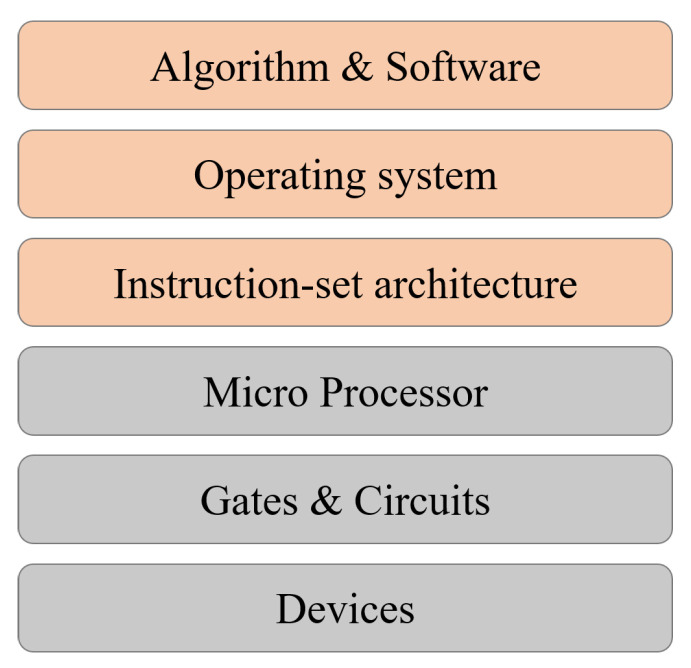
Layers of abstraction of a computer.

The power of quantum computing is currently mainly demonstrated by quantum algorithms. Given a problem, a quantum algorithm is constructed in the framework of a universal quantum computing model, such as the circuit model [6], or a Hamiltonian-based model [1,7]. An algorithm is realized by a sequence of elementary operations available in a model, such as CNOT gates and qubit gates [8]. However, for the modern design of computers [9], the above is not enough to guide the design of a real quantum computer. A computer system is far more complicated than a physical experimental device. From the hierarchy of the layers of abstraction, the physical devices and gates are at the bottom layer of the hierarchy, while algorithms and applications are at the top; see Figure 1. There is a gap between them. Some investigations on quantum high-level programming and layered design have been taken, e.g., Refs. [10,11]. Filling the gap, although it may take decades, is necessary to build real quantum computing systems.

To this end, we need to understand how to satisfy the requirements of programmability, modularity, automation, etc., as well as the basic requirement of universality. A computing device or system is programmable if it can realize a broad range of programs (or algorithms) without almost any change of its physical structure. That is, programs can be loaded as software. A system is modular if the connections between different parts of it, known as units, are device-independent, namely, a unit can be detached or replaced without affecting other units. A system is automate if it can realize hierarchical or concatenated tasks without actively interfering in the middle. Realizing these features has greatly benefited modern computers and also engineering.

With the methodology above, in this work we present a survey of quantum von Neumann architecture. There is some exploration on this subject in the literature [12,13,14]; however, it did not present a universal model with explicit stored quantum programs. Based on channel-state duality [15], a universal model for quantum von Neumann architecture has recently been developed [16,17,18,19]. In this work, we further study it by focusing on a few subjects, especially the structure of the quantum CPU, also known as QPU, and the structure of the quantum control unit (QCU). We also survey the elementary requirements for a near-term demonstration of this architecture. Our study is purely theoretical without referring to any actual quantum computing platforms. With this survey, we hope to explain some details of the model and identify some research directions to investigate in the near future.

This work contains the following parts. In Section 2, we first review the principle of classical computers. We then review the basics of quantum computing in Section 3. We then survey the basic operations in quantum von Neumann architecture (QvN) in Section 4. After this, in Section 5, we discuss the features of our model compared with other quantum computing models. In Section 6, we survey algorithm designs in QvN and their possible computational advantages. We then study the basic requirement for an NISQ implementation of QvN in Section 7. We then conclude in Section 8 with open questions and perspectives.

## 2. Classical Computers

It would be interesting to review how a classical computer is built, mainly regarding their underlying principles. This will help to understand the current situations for quantum computing. In this section, we start from a few universal computing models and then move on to the layers of structures for the design of a modern computer.

### 2.1. Computing Models

A universal computing model is a framework to design algorithms for solving problems. The most well-known model is the circuit model based on Boolean logic, while at the same time there are a few equivalent ones, including the Turing machine, cellular automata, etc. Their logic building blocks are different but can simulate each other efficiently [5].

We start from the circuit model. Information or data are represented as bit strings, and the basic Boolean gates on bits include NOT, AND, NAND, OR, etc. An important theorem is that there exist a universal set of gates so that any Boolean function, $f:{\left\{0,1\right\}}^{n}\mapsto \left\{0,1\right\}$, can be expressed as a sequence of these gates, forming a circuit. Such circuits are not invertible as some bits are lost, but they can be made invertible by using the Toffoli gate to simulate them. The Toffoli gate is

(1) $$\mathrm{Tof}=P_{0}⊗1+P_{1}⊗\mathrm{CNOT},$$

for the controlled-not gate

(2) $$\mathrm{CNOT}=P_{0}⊗1+P_{1}⊗\mathrm{NOT},$$

with $P_{0}$ ($P_{1}$) as a projection on bit-value 0 (1). Despite this, a circuit is often designed using the Boolean gates.

The circuit model is fundamental for the characterization of universality and also the design of algorithms. However, it is still abstract without specifying components for building a real computer. The foundation for the design of modern computers is the so-called von Neumann architecture (vNA) [20], which contains a few modular parts, including the central processing unit (CPU), memory, control, internet, and in/out units. All these can be described by the circuit model, but it is crucial to separate them. In particular, the stored programs in the memory unit are essential for realizing universality and programmability. Namely, a stored program as bit strings can be read and then loaded to the programmable CPU without physically changing the structure of the CPU in order to run different algorithms (i.e., programs). Formally, this realizes

(3) $$G\left(\vec{b}\times {\vec{b}}_{A}\right)=A\vec{b}\times {\vec{b}}_{A}^{\prime },$$

for *G* as the CPU, $\vec{b}$ as an input bit string, and ${\vec{b}}_{A}$ as the bit-string encoding of an algorithm *A*. The desired output is $A\vec{b}$. The final ${\vec{b}}_{A}^{\prime }$ is often ignored but can be used to recover ${\vec{b}}_{A}$. The program also contains control signals for precise timing and the addressing of data and operations or commands. Although the internet was invented later than the vNA itself, and there are also many types of communication, the download and upload of data is an indispensable part of vNA.

Although it seems vNA is a step closer to a real computer than the circuit model itself, vNA is still an abstract model. A modern computer is far more complicated than the abstraction of vNA. In particular, it follows a hierarchical design of layers of abstraction, with the physical logic devices at the bottom and algorithms and applications at the top. For instance, there are many types of memory, such as internal storage, hard disk (as external storage), and flash memory, etc., playing distinct roles in computers and also microchips.

### 2.2. Hierarchical Design

The hierarchical layers of abstraction for a computer architecture is a crucial step to build a real universal programmable computer [9]. Here we take a brief overview of it, mainly the physical aspects; see also Figure 1. It contains both hardware and software layers, with different programming languages associated with them.

- The physical devices: a physical system used as the carrier of bits. For instance, they are the transistors as the basic element to construct logic gates, or the magnetic domain for storage.
- The gates and circuits: used to design the elementary Boolean gates and also elementary circuits, such as the adder, multiplexer, decoder, and a few sequential circuits, such as Latch, Flip-Flop, and Register. This takes place on the level of machine language.
- The micro-processor: based on vNA. It can realize instructions such as if, else, when, while, for, shift, branch, etc., for the purpose of programming.
- The instruction-set architecture: to design instructions, operand locations, memory, control, etc., such as CISC and MIPS. This is on the level of assemble language.
- The operating system: decides how people can use a computer, such as how to input/output and how to input commands.
- The algorithm and software: programs for solving a certain class of problem. This is on the level of advanced language.

From the hierarchy of the layers of abstraction, we can see that quantum computing is still at an early stage. Currently, what people mostly aim at is a quantum CPU that can run simple circuit-level quantum algorithms, while all other parts can be classical. Namely, it uses classical control, classical memory, and also classical operating systems. The quality of qubits and also circuits are getting better, but these are at the lower levels of the hierarchy. There is no real logical qubit yet, which shall be error-correcting, either self-correcting or actively. It is still at infancy to construct a quantum micro-processor and instruction-set architecture, and this requires a better understanding of the roles of quantum memory and quantum control, and the roles of being quantum in other devices.

## 3. Basics of Quantum Computing

In this section, we briefly review the basics of quantum computing [8] and set the stage for our study. We focus on finite-dimensional Hilbert spaces. For a Hilbert space, *H*, quantum states are known as density operators, ρ∈D(H), forming a convex set of non-negative semi-definite operators with trace 1. A state is pure if it is also a projector. Quantum evolution is in general described by completely positive, trace-preserving (CPTP) maps [21], known as quantum channels. A fundamental principle is the quantum channel-state duality, i.e., the Choi-Jamiołkowski isomorphism [15,22], which maps a channel, E, into a quantum state:

(4) $$\omega_{E}:=E⊗1(|\omega \rangle \langle \omega |),$$

called Choi state in this work, for

(5) $$|\omega \rangle :=\frac{1}{\sqrt{d}}\sum_{i=0}^{d-1}|i,i\rangle \in H⊗H$$

as a Bell state, known as an ebit, with d=dim(H).

A channel can also be written as a Kraus operator-sum representation

(6) $$E\left(\rho \right)=\sum_{i=1}^{r}K_{i}\rho K_{i}^{†},$$

for $K_{i}$ as Kraus operators [21] with $\sum_{i}K_{i}^{†}K_{i}=1$. This can be found from the eigen-decomposition of $\omega_{E}$, and *r* is the rank of $\omega_{E}$.

Unitary evolution and quantum measurement can both be viewed as channels. A unitary U∈SU(d) is rank 1, with $U^{†}U=UU^{†}=1$. Its dual state is a pure state, $|\omega_{U}\rangle =\left(U⊗1\right)|\omega \rangle$, and we will use the notation |U〉 for simplicity. A quantum measurement is a POVM (positive operator-valued measure), which is a set of positive operators, $\left\{M_{i}\right\}$, with $\sum_{i}M_{i}=1$. It is clear to see each effect $M_{i}$ can realize, such as $M_{i}=K_{i}^{†}K_{i}$ for a Kraus operator, $K_{i}$; therefore, the POVM is realized by a channel.

A channel can be described as an isometry *V* with $V=\sum_{i}|i\rangle K_{i}$, so it can be realized by a unitary *U* with V=U|0〉 as the first block column of *U*, and |0〉 as the initial ancillary state. This is the Stinespring’s dilation theorem, which guarantees that it is enough to consider unitary evolution on pure states, since non-unitary channels and mixed states can be realized by ignoring ancilla or subsystems.

In quantum circuit models, we consider unitary evolution on multi-qubit states followed by measurement. Similar with the classical circuit model, there also exist universal gate sets to decompose arbitrary unitary operator [23]. Two well-known examples are the sets {H,T,CNOT} and {H,Tof}, for the Hadamard gate, H, and the T gate as the forth-root of the Pauli Z operator. The Toffoli gate is universal for classical computing, but with the H gate, they are universal for quantum computing. The H gate exchanges Pauli X and Z operators:

(7) HXH=Z,HZH=X,

while with $T^{2}$, which is the phase gate S, and the CNOT, they form the Clifford group [24] that preserves the set of (tensor-product of) Pauli operators. It is known that Clifford circuits are not even universal for classical computing. Non-Clifford gates such as T and Tof are necessary to achieve quantum universality.

We see that quantum measurement is needed to read the results, which can be viewed as the expectation value of observable on the final state. It is also possible to encode expectation values as bit strings and to require the final state of quantum algorithms to be bit strings, such as using the amplitude amplification algorithm [25], but this will cost more quantum resources. To estimate expectation values, we often run the same circuit multiple times to obtain the necessary probabilities. Namely, to measure tr(Aρ) for a hermitian observable *A* on the final state, ρ, the eigenspectrum of $A=\sum_{i}a_{i}\left|i\rangle \langle i\right|$ is needed, and probabilities $p_{i}=\langle i|\rho |i\rangle$ are obtained by repeated measurments so that

(8) $$\mathrm{tr}\left(A\rho \right)=\sum_{i}p_{i}a_{i}.$$

That is, there are two primary but fundamental differences between the classical and quantum cases: the quantum evolution is unitary but non-unitary measurement is needed for readout. It is more appropriate to treat quantum algorithms as extensions of probabilistic algorithms, which not only use Boolean circuits acting on bits but also random numbers, in the form of pbits. Qubits can be understood as a combination of bits and pbits in the sense that the basis for a Hilbert space are bits, while its ampitudes on this basis are the source of pbits.

## 4. Basics of Quantum von Neumann Architecture

In this section, we discuss the basic model of quantum von Neumann architecture (QvN) based on our recent work [16,17,18,19], and here we aim to explain the details of the elementary operations in our model. Note that we do not study how to physically construct or encode a logical qubit, or physically construct a unit, which are separate important subjects.

### 4.1. The Basic Model

**Figure 2:**
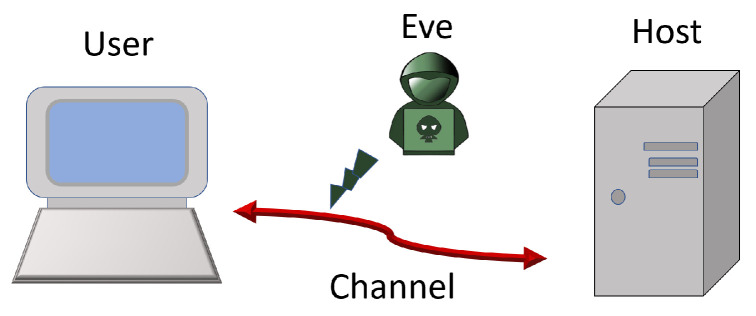
The model we use for quantum von Neumann architecture in this work. The user aims to perform a quantum algorithm, while the algorithm or program is provided by the host through a quantum channel, which can be monitored by an eavesdropper Eve.

We describe the basic model as shown in Figure 2. This is the analog of what exists nowadays for modern computer system. Of course, we only discuss the primary abstract process. A user aims to perform a quantum algorithm, while the algorithm or program is provided by the host through a quantum channel, which can be monitored by an eavesdropper, Eve, or suffers from noises. Quantum codes are needed to protect information against noises and Eve, and they are also needed for the computers.

In practice, a host or data center may have a different design from a desktop computer. However, for simplicity we assume a quantum host follows a similar design to a quantum computer. The program may come from a host or another user. Without digging into the structures of a user or host computer, below we explain the elementary operations that need to be performed.

### 4.2. Read and Write on Memory

Given a quantum program encoded in a quantum state, one has to execute it. Using Choi state, the underlying scheme is that the action of channel E on state ρ is recovered as

(9) $$E\left(\rho \right)=d\;{\mathrm{tr}}_{B}\left[\omega_{E}\left(1⊗\rho^{t}\right)\right]$$

for $\rho^{t}$ as the transpose of state ρ. The partial trace, ${\mathrm{tr}}_{B}$, is on the 2nd part of $\omega_{E}$. Below and most of the time in this work, we only consider unitary programs. A program, *U*, is stored as its Choi state, |U〉=U⊗1|ω〉; see figure:


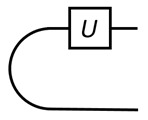


The curve is the Bell state, |ω〉. Given |U〉, how to use it? The basic usage is how it acts on input. In our scheme, an input state is injected by a binary projective measurement, and the output is also obtained by a projective measurement (PVM).

Suppose the initial state is |0〉, and we need to obtain

(10) $$p_{i}=|\langle \psi_{i}{\left|U|0\rangle \right|}^{2}.$$

The binary PVM for initial-state injection is $\left\{P_{0},P_{\bar{0}}\right\}$ for $P_{\bar{0}}=1-P_{0}$. The PVM for readout is $\{|\psi_{i}\rangle \langle \psi_{i}|\}$. As measurement outcomes are random, the initial state is only realized with finite probability. However, this is not a problem. For the case of $P_{0}$, we obtain $p_{i}$. For the case of $P_{\bar{0}}$, we obtain $p_{i}^{\prime }=1-p_{i}$, so $p_{i}$ can also be obtained [16].

If the dimension of *U* is *d*, then we need a qubit-ancilla to realize the binary PVM. See the figure for *n*-qubit input:


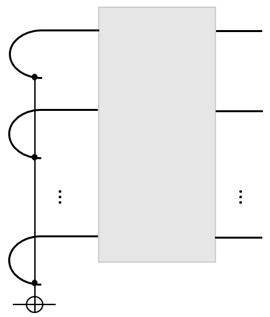


The Toffoli-like gate (on the left) is needed to extract the parity to the ancilla. A PVM on the ancilla will realize the initial-state injection. For convenience, we often call the 2nd part of a Choi state the ‘tail’, which serves as the ‘port’ for the initial-state injection, a ‘write’ operation, and the 1st part as the ‘head’, which is the port for the ‘read’ operation.

Besides the Choi-state form, there are also other ways to store a program. Note that a program, *U*, can be decomposed as a circuit of elementary gates $U\approx \prod_{i}U_{i}$, with a fixed accuracy, ϵ. Here we discuss a few of them.

- Quantum encoding: uses the Choi state |U〉.
- Classical encoding: uses bits [U] to represent *U* as a matrix, or as a sequence of gates forming a circuit, with the location and type of each gate encoded by bits.
- Hardware encoding: a gate is stored in a hardware device, just like the optical elements in photonic quantum computing [8].

Different schemes can be applied in different settings. They will also affect the construction of the QPU. Note there are also other ways. There is a non-local Choi-state-like form, which allows a program to be executed blindly [26], but this requires significantly more resources; therefore, we do not study this form in this work. Classical encoding, [U], is the most popular nowadays. It can be used for classical control signals to guide the execution of gates. This applies to the current framework on circuit models, such as superconducting circuits. Below we will study how to use quantum encoding to construct QPU.

### 4.3. Teleportation

Teleportation has been used in many ways, e.g., in quantum communication, in fault-tolerant scheme, and in measurement-based quantum computing. For QvN, teleportation is used for both communication and computation. In communication, it has been well established that teleportation can replace the transmission of qubits by bits given distributed ebits [27]. For computation, teleportation is employed to realize gate operations, similar to the measurement-based model. Here we recall its definition and motivate the covariant teleportation.

One often starts from a bipartite non-local setting that Alice and Bob already share ebits, and Alice aims to send qubits (or qudits) to Bob without quantum communication. The scheme is shown in the figure:


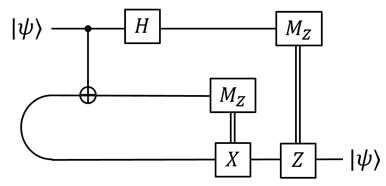


The Pauli byproducts, $\sigma_{i}\in \left\{I,X,Y,Z\right\}$, are corrected by sending the measurement outcomes, *i*, from the Bell measurement of Alice to Bob.

There is an interesting symmetry to this scheme. Each Pauli byproduct is obtained with the same probability. The operators, $\sigma_{i}$, form a projective representation of the group $Z_{2}\times Z_{2}$. Actually, this fact has been used to define group-based teleportation [28].

This can also be understood from the point of view of tensors. The set of Pauli byproducts form a three-leg tensor, and it has full symmetry, SU(2), if the identity operator is absent [29]. This also applies to any SU(d) and leads to covariant teleportation [16] by grouping the non-trivial Pauli byproducts together, namely, using a qubit ancilla to extract the binary distinction of byproducts.

### 4.4. Switchable Composition of Programs

Covariant teleportation, also called universal quantum teleportation (UQT), can be used to compose two programs together. Namely, two program states, |U〉 and |V〉, can be composed together deterministically to be |UV〉 or |VU〉, depending on the direction of information flow; see the figure:


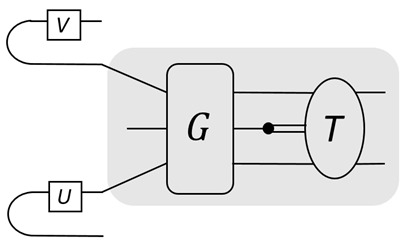


The shaded region is for the UQT. It requires a qubit ancilla and Toffoli-like gate, and also the adjoint form *T*, also known as the affine form, of a gate [16]. For instance, a qubit gate U∈SU(2) corresponds to an orthogonal rotation R∈SO(3). A PVM on the qubit ancilla leads to either trivial or non-trivial Pauli byproducts, on which the correction *T* is applied. Note that in order to complete composition, the programs need to be known, i.e., as white boxes. This can be used to generate large programs from smaller ones. When only elementary programs are composed, such as |H〉, |T〉, |CZ〉 for the Hadamard gate, H, T gate, and CZ gate, only the adjoint form of H and T needs to be done. As H exchanges Pauli X and Z, while T can generate superposition of Pauli X and Y, it is easy to see that the affine form of H is a swap gate, while T is a Hadamard-like gate [16].

The ebits used in the composition have a unique feature. A state injected at its tail can propagate ‘backward’ to its head, following from the channel-state duality. This leads to a switchable construction of the composition. For instance, for a qubit program it attaches one ebit to it. It then applies a few CZ gates, as shown in the figure


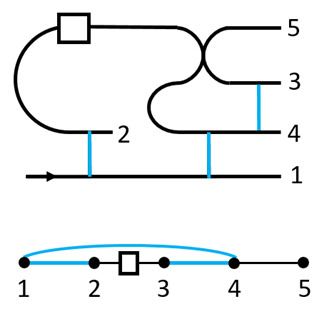


The top panel shows the circuit, while the bottom panel shows the operations on the qubits explicitly. The blue lines are CZ gates. The box is the stored program. This forms a ‘pre-compose’ step between the previous program and the current one. There are then two possible paths for the information flow, one with the current program, 1→2→3→4→5, the other without it, 1→4→5. This serves as a switch for turning the gate on or off, depending on the control signal. To complete the composition, one path needs to be chosen while closing the other, and there will also be correctable Pauli byproducts after the composition. This will be used to construct the QPU.

### 4.5. Program Conversion

It is also useful if one program can be changed into another. This needs the operation of a quantum superchannel [30,31,32]. For notation, we use a hat on the symbols for superchannels. The circuit representation of a superchannel is

(11) $$\hat{S}\left(E\right)\left(\rho \right)={\mathrm{tr}}_{a}V\;E\;U(\rho ⊗|0\rangle \langle 0|),$$

for ρ∈D(H), U and V are unitary, and a is an ancilla. The dimension of *V* can be larger than *U* [33], but we do not need the details here. This can also be represented as the action on Choi state with

(12) $$\hat{S}\left(E\right)\left(\rho \right)={\mathrm{tr}}_{\bar{A}}V⊗\tilde{U}\left(\omega_{E}⊗\omega \right)(1⊗\rho^{t}⊗|0\rangle \langle 0|).$$

The unitary $\tilde{U}$ is the transpose of U conjugated by a swap. The trace is over the subsystems, except for the top one, A. We see that ebits are needed in order to realize non-trivial superchannels; see the figure


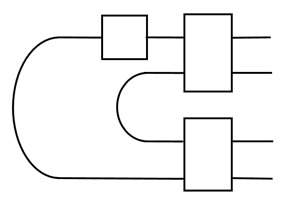


The top wire carries the output. It has been shown that [17] a sequence of superchannels acting on Choi states can be composed together with the tool UQT. This realizes the so-called quantum comb, which is a composition of superchannels. This has found applications in quantum estimation, learning, optimization, etc., and we will study this further in the Section 6.

### 4.6. QCU

Control plays an essential role in classical computers. The simplest example is the CNOT gate, which uses a bit to control another bit. Clock is another notable example and is a building block in classical sequential circuits. There are also schemes that use electric circuits to achieve control of analog signals. Here we analyze the construction of a quantum control unit (QCU) for QvN.

First, there are different layers of control tasks. The most familiar one is to use bits to control quantum gates. In the circuit model, each gate has a definite spacetime location, i.e., when and where it acts on qubits. Such classical information serves as bits to control the execution of quantum gates. There is no entanglement between the control bits and data qubits.

One semi-classical scheme is to use lasers to interact with qubits, which is a seminal field of AMO physics and also the most familiar paradigm of quantum control. There is no entanglement between the qubits and the lasers. Dynamical decoupling [34] is a notable example.

One can also use qubits to control quantum gates. This actually has been a quite common scheme for designing quantum algorithms, such as the swap test, DQC1, and also quantum phase estimation [18]. This can lead to interference of quantum gates, and this has been used in the linear combination of unitaries (LCU) algorithm [35,36] and also in a model of contextual quantum computing [18].

Using QCU also leads to certain issues. A first non-trivial issue arises if the target quantum gate is unknown, i.e., a black box. This applies to situations of modular design, for instance. It was proven that quantum control over an unknown gate is impossible [37]. This is because the operation U↦ΛU is not valid as it converts the unphysical global phase of *U* to a local phase of *U* in ΛU. Here, ΛU is the controlled-*U* gate. A solution for this is to know the eigenstate of *U*, which serves as a ‘flag’. The following circuit realizes the desired quantum control:


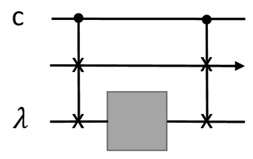


The top wire is the control register, the second wire is the data register, and the third one is the flag. Now the gate is ‘gray’ instead of black since the eigenstate and eigenvalue of it are known.

This method can be used to run quantum control over unknown programs, and then realize LCU algorithms. We require that each program state, |U〉, is given with a flag, $|\lambda_{U}\rangle$. A flag state can be injected using our initialization method; see the figure for the linear combination of two unknown programs:


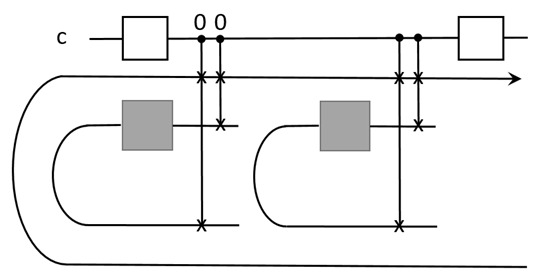


The control signal (c) itself is not pre-stored, although it can be quantum. That is, the control signal is taken as deterministic input and is not injected by measurement, which is random. Now the question is, can it also be pre-stored as quantum states? For comparison, random input data induced by PVM is acceptable since these input signals are orthogonal and their results are effectively equivalent. A random control signal induced by PVM will lead to uncertain operation, say $U_{1}$ or $U_{2}$, which are not orthogornal in general. It seems there is no easy solution to this. In order to make orthogonal control signals, the non-local scheme [26] can be used, which turns a set of $\left\{U_{i}\right\}$ into approximate orthogonal states, but this requires significantly more quantum resources. Therefore, we do not require pre-stored quantum control signals.

For all the above, the control unit is required to be separable from the data unit at the output. This is necessary since, by assumption, the control unit shall not carry the final results. They can become entangled during a computation, but at the end they will be disentangled. This issue has been analyzed in the setting of quantum Turing machines [38,39,40,41,42], and also in a recent study of quantum control machines [43]. For instance, in the model of a local quantum Turing machine [42], by expressing the final quantum state carrying the results as a matrix-product state,

(13) $$|\psi \rangle =\sum_{i}A\left(i_{n}\right)\cdots A\left(i_{2}\right)A\left(i_{1}\right)\left|\ell \rangle \right|i_{n}\cdots i_{2}i_{1}\rangle ,$$

the machine register, with an edge state, |ℓ〉, serving as the control register, can be disentangled from the data register at the end. Another method is to use measurement feedback to disentangle the controller and data [18] used to define a contextual quantum computing model. These examples also show that, due to entanglement, the interplay between the control flow and data flow needs more study.

### 4.7. QPU

A CPU usually contains a control unit and an ALU (arithmic logic unit). For the classical case, to run a program, *A*, which is stored as bits, [A], in the hard disc, the [A] is firstly loaded as control and operations on a programmable circuit, aka., a chip. The internal storage is also used to store temporary data. In this section, we study the primary structure of the QPU in our model, and compare it with existing ones.

For the quantum case, the starting point is the circuit model. However, there are different approaches. We find there are two dual ones:

- Type-I: gates are stored as hardware while qubits are sort of ‘not there’; this applies to linear optics, which uses optical elements as gates and photons as qubits;
- Type-II: qubits are stored as hardware while gates are sort of ‘not there’; this applies to SC, trapped ions, which uses laser pulses (interacting with matter particles) as gates and particles (e.g., electrons) as qubits.

For both of these, the program, [U], that is used as control signals is classical. We call this ‘classical programmability’. On the contrary, we will define a quantum programmability for our model of QPU. It relies on the quantum encoding of programs, or a semi-quantum one, namely, Choi states |H〉, |T〉, and |CX〉, to store the elementary gates, or other Choi states to store blocks of gates, and bits [U] to store their spacetime locations. Furthermore, we need the toolboxes of switchable composition, quantum superchannels, and also quantum control units.

We have seen that the control flow is distinct from the data flow. Actually, control sequences can also be stored as programs, but in order to compose them, another level of control is still needed as long as the QPU is not automatic. That is, all control signals are needed to monitor the evolution. As discussed in the former section, the input control signals need to be deterministic. That is, the [U] is used as the control signal to apply composition and other operations on primary Choi states. To run *U*, qubits in the QPU will be measured. After the run, qubits need to be refreshed to the right Choi states.

For instance, consider the programmable realization of a sequence of H and T gates to approximate a qubit rotation; see the figure


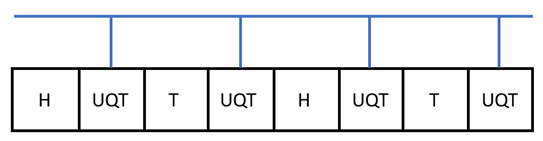


The UQT serving as the switchable composition is under control. The elementary programs need to be refreshable in order to be multi-time programmable. Furthermore, the QPU can also perform superchannels on the programs. These can lead to quantum superalgorithms that prove to be powerful. We will discuss this more in Section 6.

The ‘programmability’ in our model is not classical. There are a few aspects for our programmability, referring to the memory unit and control unit. First, our model allows programs to be stored as quantum states, i.e., Choi states. This means the algorithms in our model can use quantum program states. Second, a stored quantum program is switchable, on or off, depending on the control signal. Third, the control signal can also be quantum, so it forms an important part of a quantum algorithm.

Our construction of the QPU lies somehow between type-I and type-II above. We have named this type-III; see Table 1. For type-I, gates are stored as hardware, so they exist in the space domain, while qubits such as photons are only generated on demand when an algorithm is to be run, so they exist in the time domain. It is the opposite for type-II. For type-III, qubits are not used to carry data but are used to encode programs. The actual data qubits are prepared by measurements when an algorithm is to be run, so they exist in both the space and the time domain. A gate also exists both in the form of a program and the operation in a composition, and so in the spacetime domain.

What is the advantage of using type-III? At present, this is not fully clear. It apparently consumes significantly more qubits to realize an algorithm due to the usage of teleportation, as in the case of the measurement-based model [44]. That said, teleportation brings some advantages (see Section 4.3). For instance, for the architecture design, the physical qubits can tolerate more decoherence since they only carry the data for depth one before a composition occurs. In addition, only qubits need to be manufactured, and gates are applied to them. Finally, there is a clear resource-theoretic characterization of QvN, by treating quantum memory as a universal resource [18]. This can benefit the understanding of quantum superalgorithms, which rely on quantum memory.

### 4.8. Program Download/Upload

After a computation, the program state is consumed/destroyed. This is not a flaw, however. This is also present in the usual quantum circuit model: after the computation, the qubits need to be refreshed for the next task. This even exists in classical computers that use temporary data such as cache and buffer.

**Figure 3:**
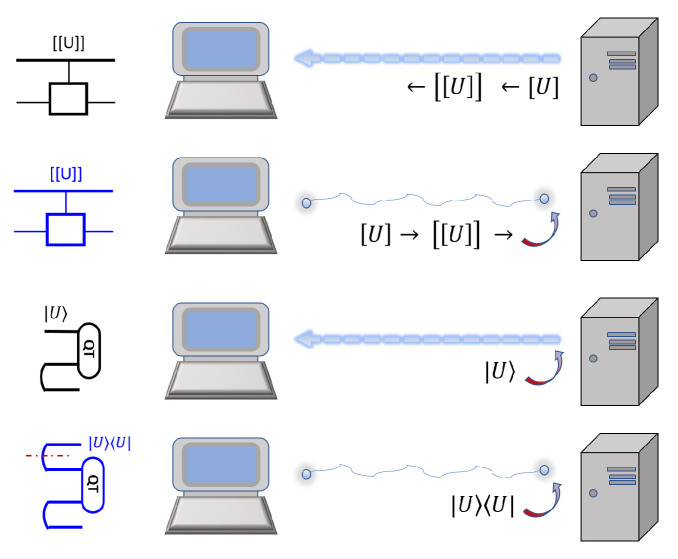
The four basic schemes to realize the download of quantum programs. The upload would be similar.

For QvN, the program states are likely stored in the memory unit. A computation would basically turn some of the states into garbage, and the user would have to restore the programs. This is achieved by downloading them through the internet from a software producer, or a host. In order to be secure, here we consider quantum internet via quantum communication. The usual carrier, although it does not have to be, is photons. Therefore, the user needs to have the ability to receive photons and to store and measure them. We find, as shown in Figure 3, there are four primary schemes:

1. Use qubits to send bits: the host employs the bit-string description, [U], of a program, *U*, and then encrypt the bits, [[U]]. Quantum cryptography such as the BB84 scheme can be used to send the bits [45]. At the user side, a control device is needed to receive the bits and apply the gate sequence, without revealing the bits to the user. This is very much like a delegated computing or remote state preparation [46], but now the user is the remote site, and the host does not need to verify the user.
2. Use ebits to send bits: one can use ebit-based quantum cryptography to send bit-string descriptions of the gate sequence [U].
3. Use qubits to send qubits: the host prepares photons at the state |U〉 directly, and sends them to the user, who then applies quantum teleportation between photons and the memory qubits to teleport/download the program from the photons to the memory qubits.
4. Use ebits to send qubits: the host and user first establish many pairs of ebits of photons, and then the user applies quantum teleportation between some photons and the memory qubits to teleport/download the program from the photons to the memory qubits. Namely, if |U〉=V|0〉, the host applies $V^{t}$ and then the projection |0〉〈0| on his side, and that will prepare the photons at the user as |U〉. The host needs to use our initial-state injection technique, and the effect on the final readout at the user’s side can be easily dealt with.

One may wonder which scheme is preferred. For the first two, the goal is to send bits, which need to encode both the space and time information of the gate sequence in a program. For the last two, the goal is to send qubits, which does not need to encode the time information, hence consuming fewer numbers of qubits than the number of bits. However, currently qubits are much more expensive than bits. The choice of a scheme would depend on many practical conditions.

### 4.9. Program Verification

When a user decides to download a program from a host, the user has to verify that the host indeed has the promised program. This is a quantum verification task [47,48], which has been widely studied in recent years. Here we discuss how the program-verification would work, but we do not specify all the details since there could be various schemes depending on practical settings.

The verification can be interactive. In the framework of interactive-proof systems [47], the user serves as a verifier and the host serves as a prover. Usually, the verifier is required to be computationally in BPP, while the prover is in BQP. However, here in our setting the verifier is also in BQP, but they only have a limited number of copies of the unknown program |U〉. That is, the user, as the verifier, can only perform verification instead of a full tomography.

It is not hard to determine the number of samples of |U〉 the user can download. From verification theory, which specifies an infidelity parameter, ϵ, and confidence parameter, δ, the number of samples scales as

(14) $$N\in O\left(\frac{1}{\epsilon }\log\frac{1}{\delta }\right),$$

ignoring other factors that do not matter for our discussion here. Although the scaling with respect to ϵ is not efficient, for the purpose of verification a moderate fidelity is acceptable, and the confidence is usually more important.

Given a few samples of |U〉, the user can also perform quantum estimation or learning. It is also well established that the fidelity scales as $N^{-2}$ for optimal joint global operations on them [49]. This is the so-called Heisenberg limit.

For a full process tomography, the user has to use a number of measurement operations, hence a number of samples, that scale with the dimension of |U〉, which is exponential with the number of qubits it acts on. Therefore, we find that as long as the number of samples is much smaller than that for tomography, the verification can be carried out efficiently. In addition, there is also another level of sampling, which is to obtain the final expectation value of the observable. In modern terms, this is a special instance of shadow tomography, which can be performed with a small number of samples [48].

Verification is an important subject in the study of blind or delegated quantum computing. We will study its difference from QvN in Section 5.3.

## 5. Difference from Other Models

In this section, we analyze the primary differences between QvN and some other models.

### 5.1. Circuit Model

Here we compare QvN with the quantum circuit model (QCM), including the execution of an algorithm, security, verification, and other issues. We assume the usual scheme of QCM, which realizes a quantum algorithm as a three-stage process: initial state preparation, gate execution, and measurement. We already see their difference from our study of programmable QPU.

Actually, it is also fine to treat QvN in the framework of QCM, as the primary operations are either unitary gates or measurements. However, it is necessary to make a distinction between them since conceptually QvN considers more requirements. This is similar for the classical case.

The scheme to realize a circuit can be seen from this figure:


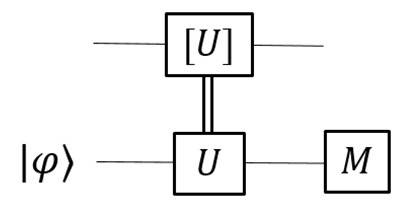


The top register is classical, and [U] is the classical representation of the program *U*. The output from a quantum algorithm is assumed to be the expectation value of a hermitian operator, which reduces to the estimation of a set of probabilities, $p_{i}$. The implementation of a quantum algorithm can be performed efficiently provided that:

- the initial state can be prepared quantum efficiently;
- the program *U* can be stored classically efficiently, as [U];
- the program *U* can be realized quantum efficiently;
- the measurement for readout can be realized quantum efficiently;
- the number of samples scales efficiently to estimate each $p_{i}$.

The classical description, [U], is often a bit-string of the gate sequence in the circuit, if $U=\prod_{i}U_{i}$, while bits are used to encode the spacetime location and type of each primary gate, $U_{i}$, from a universal gate set, e.g., {H,T,CZ}.

After the execution, all qubits are measured and need to be refreshed for further usage. The program is stored as bits, [U], so do not need refreshment. The composition of two programs, $U_{1}$ and $U_{2}$, is simple, namely, they are implemented sequentially. The initial state needs to be prepared before the application of gates, which is not the case for QvN.

Another notable difference is that QvN requires the download of quantum programs, since they cannot be cloned if the bit-string descriptions of them are unavailable. The security of quantum communication, relying on the uncertainty principle, ensures the security of the quantum programs. For the circuit model, a circuit is often not secure, i.e., there is a classical circuit *diagram*, which can be easily seen and copied. However, there are also secure protocols relying on QCM. A notable example is blind or delegated quantum computing (DQC) [50], which was initially formulated via MBQC but can also be formulated via QCM. This leads to the discussion in the following two subsections.

### 5.2. MBQC

Besides the circuit model, QvN also has close connections with MBQC. In the standard MBQC, also known as the one-way model [44], a resource state such as the 2D cluster state is given, and then a sequence of local adaptive measurements is performed to execute the gates. The basic underlying mechanism is 1-bit teleportation [29], and a spatial direction is chosen as the ‘teleported’ evolution direction. Furthermore, it is equivalent to the model based on Bell measurements for standard teleportation, which is 2-bit [51]. Here we will denote this as the teleportation-based model (TBQC), although sometimes it is treated as a special case of MBQC.

For clarity, we have summarized the comparison in Table 2. The TBQC is often used for the fault-tolerant execution of gates; hence, its byproduct is extended to Clifford operations, which still preserve Pauli gates. Due to the covariant teleportation used in QvN, the stored program can be fully quantum, namely, the whole group, SU(d), of gates. On the contrary, in MBQC qubit gates are induced by local measurements in rotated bases, while in TBQC the entangling of gates such as CZ or CNOT is performed by two parallel Bell measurements, consuming ebits. One should note that in MBQC the local measurements are relatively simple, compared with Bell measurements, and the resource state can be prepared offine.

**Figure 4:**
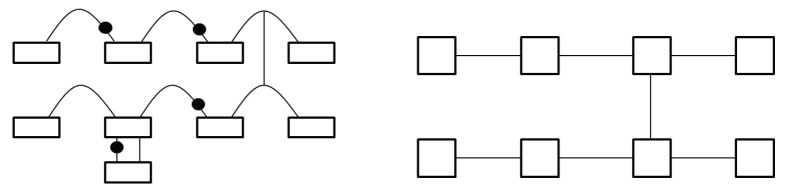
Information flow in QvN (**left**) and MBQC (**right**). The vertical line represents the CZ gate. For QvN, a box represents a composition, which is a measurement for teleportation. The curve with a dot represents a qubit program. A switchable program is also shown. For MBQC, a box represents a measurement on a site that realizes teleportation.

Another important difference is that in QvN the information, injected at the ‘tail’, is always carried by the ‘head’ of a Choi state. There is no such explicit head–tail structure for MBQC, or for TBQC. The information flow is shown in Figure 4. For QvN, the information never ‘crosses’ a composition ‘box’, but this is the opposite for MBQC. Treating a composition as a single depth, each physical qubit for a tail or head only has depth one. This leads to the switchability of the composition, also illustrated in the figure. As has been discussed, the switchability could be useful to construct the QPU.

### 5.3. Delegated Quantum Computing

An important model for secure computation is delegated quantum computing (DQC), which was earlier known as blind quantum computing [50]. In this model, a user, as a verifier, aims to delegate computation to a prover without revealing the computation to the prover, see the figure for QCM in Section 5.1, while the classical and quantum registers belongs to the verifier and prover, separately. The verifier is in BPP, while the prover is in BQP. Usually, the verifier knows what to compute but does not have the capability to do so. This model may apply to the recent era of quantum computing, where only a few labs or companies have powerful quantum computers, and customers can use them blindly and confidently. The input, output, and the computation itself can all be blind to the prover.

This is different from the program-verification in QvN. For QvN, the host has both [U] and |U〉, while the user only has |U〉. The user will use the program blindly by making measurements. Given the limited samples of a program, the user can not perform tomography, i.e., cannot obtain its classical description, [U]. In DQC, the prover can do *U*, which is equivalent to the ability to prepare |U〉, while the verifier has [U]. In QvN, the user side is BQP and the host/prover is also BQP. The purpose of verification in DQC is to verify [U], while the purpose of verification in QvN is to verify |U〉. There is no apparent delegation in QvN; see Table 3.

## 6. Quantum Algorithms in QvN

In this section, we study the design of quantum algorithms in QvN. This has been analyzed in our previous work [17,52], while here our discussion will be more specific, drawing the connection with computational advantages.

### 6.1. Quantum Superalgorithm

A quantum algorithm is usually specified by a quantum circuit and a measurement procedure, as has been shown. On top of that, there is also a classical algorithm that designs the quantum algorithm; see the figure:


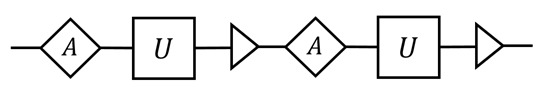


Here, a triangle represents a measurement. This design can be iterative, with measurement outcomes fed forward to the classical algorithm, *A*, which then optimizes the parameterized quantum circuit, *U*. Some examples are the Solovay–Kitaev algorithm for gate compiling [53], quantum channel simulation [33,54], and quantum approximate optimization [55]. This actually forms a classical comb of classical-quantum hybrid algorithms, using the terminology of quantum superchannel theory [30,31,32].

One can also pose the following question: can we use a quantum algorithm to design another quantum algorithm? Such a scheme works for the classical case; namely, there are classical algorithms that design classical algorithms. Such algorithms are often known as ‘meta’ algorithms or ‘hyper’ algorithms since they contain some meta or hyper variables that need to be optimized. This plays an essential role in machine learning [56].

For the quantum case, it has been confirmed that we can use a quantum algorithm to design another quantum algorithm. This follows from nothing but the quantum superchannel theory. The superchannel plays the role of the ‘meta’ algorithm, while the channels acted upon by the superchannel serve as the input to it. We will call these algorithms quantum superalgorithms to be consistent with the superchannel theory. It has the following structure:


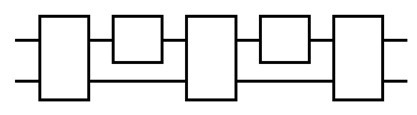


For the composition technique, it can also be realized by a sequence of compositions [17]. This is what the QPU from Section 4.7 can do. Compared with the former scheme, it is clear to see here that quantum memory (the bottom register) is used as resources to realize superalgorithms [18].

Many quantum algorithms are of this form, although sometimes they are not under the name of ‘superalgorithm’. This includes quantum estimation and learning algorithms [56], quantum channel discrimination [57], schemes for quantum games [58], quantum optimization [59], and quantum machine learning [60,61,62,63]. The recent quantum singular-value transformation [64], which can unify some quantum algorithms, is also a special type of superalgorithm [52].

The theory of superchannels also allows the so-called higher-order operations [32], which are superchannels that act on superchannels, by using the channel-state duality iteratively. These higher-order superchannels can still be viewed as a superchannel but with a more complicated multipartite structure [17].

Furthermore, one may wonder that a ‘mother’ algorithm is still needed to design a quantum superalgorithm, and such a mother algorithm can be classical. This is indeed the case, but what matters is that quantum memory, and also control, are used as resources in addition to the current framework based on the circuit model. Recently, people have found that quantum memory can lead to exponential advantages for solving some problems [63,65].

Finally, the output for the final result often contains probabilities. This requires many runs of an algorithm to estimate them. However, there is a method to convert probabilities into amplitudes of quantum states and then use the quantum amplitude estimation algorithm to obtain them in the form of bit strings [25]. This is the analog of quantum phase estimation, which can be used to estimate unknown parameters by encoding them as phase factors. Such algorithms use quantum controlled operations. From matrix decomposition, a controlled operation can be decomposed as the product of a few operations without control and simple controlled operations, such as the controlled-not gates. This means that these estimation algorithms can also be put into the framework of quantum superalgorithms.

### 6.2. Computational Advantages

Finding computational advantages would depend on the structure of certain problems. If there is no structure, quantum computing can only provide quadratic advantages. For instance, Grover’s algorithm shows that for a structure-less database of size *N*, a quantum computer will take time in order $O(\sqrt{N})$ instead of logN [8]. The so-called Heisenberg’s limit from the uncertainty principle sets the bound for the precision of estimating an unknown parameter, also with quadratic improvement of precision [66]. This relates to the fact that the square of amplitudes yields probabilities. In the model of QvN, we analyze the potential advantages by using a combination of quantum CPU, control, memory, and internet, compared to the classical case and also other quantum computing models. Our analysis, however, is preliminary and we hope this can inspire more investigations.

#### 6.2.1. Storage

It seems the amplitudes of qubits can significantly increase the ability for storage. A pure state, $|\psi \rangle =\sum_{i}\psi_{i}|i\rangle$, already stores the amplitudes, $\psi_{i}$, in it without encoding them as bit strings. However, this turns out not to be completely true, since quantum measurements are needed in order to know $\psi_{i}$, and this requires a significant amount of copies of the qubit |ψ〉.

The seminal result is the Holevo bound [67], which was established originally in the setting of quantum communication. It states that the quantum capacity of a channel is half of its classical capacity. Namely, a qubit can only be used to store or transmit two bits. This may not be hard to understand from the point of view of error correction, whereas correcting Pauli X and Z is enough to correct any linear combination of them. This is also demonstrated by the quantum teleportation and dense coding [27]. Using an ebit, a qubit can be used to transmit two bits, and two bits can be used to transmit a qubit. It also relates to the quadratic speedup of Grover’s algorithm. An *n*-qubit state can be viewed as 2n bits, hence representing $2^{2n}$ different values forming a database whose size is the square of that from *n* bits. Quantum search, if treated as a state preparation, cannot be faster since otherwise a qubit would carry more than two bits. However, this does not mean there is no larger advantages for specific tasks.

In the circuit model, a state, |ψ〉, can be represented by its preparation circuit, *U*. As has been discussed, there is an efficient bit-string encoding, [U], of the circuit, by only encoding the type of each gate and its spacetime location. However, using qubits to store quantum states can offer advantages, e.g., as we have mentioned for quantum learning [63,65]. For instance, it has been proven that, although learning the statistical average may not offer an advantage, accurately predicting the value of any observable can have an exponential advantage by using quantum memory [63]. Therefore, it is promising to explore more primacy of quantum memory.

#### 6.2.2. Speed

Quantum advantage is often used the same as speedup. This is basically because, in principle, other resource costs can be treated as a cost of computing time, but we will distinguish them since this could inspire different intuitions for solving different problems.

First, we need to distinguish an algorithmic speedup from the speed of a quantum computer. The former is on the software level, while the later also depends on the hardware. The same quantum algorithm would take a different amount of time on different quantum computers.

The underlying mechanism for algorithmic speedup relates to interference. Compared to the classical case, quantum evolution is unitary, i.e., coherent, and there are significant amounts of interference between trajectories, given a fixed basis of the underlying Hilbert space. A speedup occurs if the interference can enhance the probability of the desired trajectory. Note that the pre-condition for a speedup is the accuracy of computing result. The more accurate the results, the more time is needed.

To achieve a speedup is one of the central tasks of QPU, besides programmability. In the model of QvN, the interplay between QPU and other units will also affect the speedup. This is also the case for classical computers, and that is why cache is needed to replace the hard drive. If a truly quantum computer can be built in the future, following a certain QvN and the hierarchical design, there will certainly be various kinds of quantum memory, control, communication, and even input and output devices, etc. The speed of such a quantum computer would depend on many factors that are still hard to define at present.

#### 6.2.3. Security

Quantum cryptography to realize security was one of the prompters of quantum information science, with the BB84 scheme of quantum key distribution as a notable example [45]. It is based on the no-cloning theorem, which is equivalent to the Heisenberg uncertainty principle.

The program download/upload process can be seen as a special task in quantum communication, and it therefore shares features of standard quantum communication. Here, we should point out that it is secure in two senses. First, the program generated by the host is secure against the user. Second, the communication between the user and the host is secure against eavesdroppers. There are also other settings of secure quantum computing, such as delegated or blind quantum computing [50,68], as discussed in Section 5.3, and multi-party quantum computing [69]. For the later, neither one of the parties know the result. Instead, they must communicate to extract the result.

Attacks in quantum cryptography have been well studied [70]. An attack can be detected but not prevented. In the model of QvN, data are stored as qubits instead of bits. One may then be curious if quantum data can be hacked. Although qubit encoding data such as passwords cannot be accurately cloned and hence leaked, they can be measured. A simple quantum virus can be an instruction to make the most trivial measurement, which will erase any data. Such a virus is actually classical since it can take the same form and can copy itself. Although there are costs to make an attack possibly at any time, it cannot be prevented in principle. We therefore suppose that this stands as a ‘no-go’ for a no-no-virus hope for quantum computers. Despite this, quantum computers have the potential for more applications in cryptography due to resources such as coherence and entanglement.

#### 6.2.4. Energy

Reducing energy consumption in computation was one of the original motivations for quantum computing. Landauer showed that erasing bits will cost energy, while with Toffoli gates, a classical computation can be made reversible [71]. At the same time, it was realized by Feynman and others that a quantum computer can be reversible since its evolution is unitary [1]. However, compared with other features, the study of energy consumption in quantum computing is rare [72,73,74].

In the circuit model, initialization and readout by measurement will cost energy. A recent pioneer studied the energy cost in unitary evolution using superchannel theory and resource theory [74], which showed that the energy cost relates to the accuracy of the computation. However, a systematic understanding of the thermodynamics of unitary evolution is lacking. This is not obvious as thermodynamics often deals with non-unitary dissipative evolution.

Here, we should point out that the energy issue could be relevant for the design of quantum control schemes, compared with classical ones. However, it is not always straightforward to assess the amount of energy cost since some schemes are ‘semi-classical.’ For instance, cooling a qubit by a reservoir in order to suppress decoherence can be considered semi-classical. Using quantum error correction can also suppress decoherence, but it is not easy to compare the energy costs during the cooling and the error correction.

In the model of QvN, a quantum control unit is used to enact quantum operations and also forms the ingredients of quantum algorithms. When the control signal is not a part of the final output, erasing or resetting it will cost energy. On the contrary, using classical control cannot generate entanglement between the controller and the target. At present, it is not clear how to find quantum advantages on energy costs over classical control schemes.

## 7. NISQ Implementation

In this section, we study how to implement a small-scale QvN on noisy intermediate-scale quantum (NISQ) devices. This would not include massive quantum error correction and quantum verification, for instance, which require more quantum resources.

We can make comparisons with the basic requirements of the circuit model. This dates back to twenty years ago [75], with five requirements: a scalable system of qubits, initialization of qubits, sufficient coherence to carry out an algorithm, a universal set of unitary gates, and measurement for readout.

These requirements are also strengthened or expanded for more purposes. They are also the basic requirements to realize a QvN. A few additional requirements are needed. (1) First, it requires the ability to execute multi-qubit controlled gates, such as the Toffoli gate. Such gates can be decomposed into elementary one- or two-qubit gates, but it would be better if they could be directly realized. These gates are needed for initialization, composition, and quantum control. (2) Second, it requires quantum communication. This was also an extra request when flying qubits such as photons are needed to connect a few separate quantum stations, such as in the trapped-ions setup [8]. The above two requirements can already be satisfied by some systems [76,77,78]. Therefore, it is possible to demonstrate prototypes of QvN.

Here we describe almost the smallest system of QvN, with the process of read–write, download, composition, control, and superchannels. They are listed as follows:

- The read–write program: It needs two qubits to store a qubit gate, and four to store a CZ gate. The initial-state injection (i.e., write) for a qubit program on a standard basis (|0〉 and |1〉) does not require ancilla, which is also the case for the read operation. For the CZ program, the write operation on a standard basis (|00〉, |01〉, |10〉, |11〉) does require a qubit ancilla and the Toffoli gate, but the read operation does not.
- The download process: For the scheme using ebits to send qubits, the state-injection at the host side requires a Toffoli gate and an ancilla. For a qubit program, the teleportation at the user side is on 4 qubits. The download in total involves 9 qubits. It is easy to verify for a two-qubit program; the teleportation is on 8 qubits. The download in total involves 17 qubits. For other schemes of the download, it requires less qubits, hence also less gates.
- The composition: To compose two qubit-program states deterministically, this needs 5 qubits with one as the ancilla. To compose a qubit program with the CZ program deterministically, it needs 6 qubits if the qubit program is applied earlier, and 7 if it is applied after the CZ. However, if the Pauli byproduct is not required to be corrected, less qubits are needed. This reduces to 4 and 6, respectively. In addition, to make the composition switchable, extra ebits are needed.
- The quantum control: To realize a quantum control of an unknown qubit program, it requires 5 qubits, with one for a qubit control, two for the qubit program, and two for the data register. Recall that an eigenstate of the unknown qubit gate shall be known, and it will be injected by measurement. For the control of an unknown two-qubit program, it needs 9 qubits.
- The quantum superchannel: To realize an arbitrary qubit superchannel, it needs 6 qubits, with two for the qubit program and 4 as ancilla. However, with a convex-sum decomposition algorithm [33], two ancillary qubits can be saved.
- A quantum superalgorithm: For a simple quantum superalgorithm formed by a sequence of compositions, its cost is determined by the composition. It can also include superchannels within such a superalgorithm, then its cost will be higher. For a simple demonstration, however, the Pauli byproduct can be left uncorrected, and even the initialization can be probabilistic. This will realize a probabilistic or random superalgorithm.

We see that less than 20 qubits is enough to realize the primary operations in a QvN. Quantum systems nowadays already have far more qubits than this. Therefore, more complicated operations, such as control or superalgorithms, can also be realized.

## 8. Conclusions

To conclude, in this paper we presented a systematic survey of the recently introduced model of quantum von Neumann architecture. We placed it in the more complete picture of a hierarchical design principle of modern computers, which, given sufficient space and time, can not only realize universality, but also programmability, modularity, scalability, etc. We also briefly drew its connection with other quantum computing models and algorithmic advantages.

On the theoretical side, there are also many interesting open questions. Here we list a few of them as our conclusion.

- Types of quantum memory unit. A quantum RAM model of states was developed [79], which was faster at finding a specific state than classical ones. Such a scheme can be used for the storage of Choi program states. We mentioned that there are various types of classical memory, and also memory devices. This is not clear for the quantum case. Our scheme for the quantum programs is more like internal memory, rather than external memory, i.e., a hard disc. Although in the early days of computers, gates were indeed applied on hard memory, nowadays there is a clear distinction between internal and external memory. The role of external quantum memory still needs to be investigated.
- The roles of quantum control. As we have shown, using a quantum instead of a classical control unit will cause issues such as the entanglement between the control flow and the data flow. We have also mentioned a few tools to deal with this. However, a general principle for the design of a quantum control unit is still needed. Meanwhile, specific examples and application settings are also needed to show the necessity of it over a classical one. We pointed out that energy consumption may relate to quantum control by studying the dynamics of work, heat, entropy, etc, i.e., the thermodynamics of quantum computing.
- Quantum ‘sequential’ circuit. A large class of classical circuits are known as sequential circuits, which, roughly speaking, are circuits with memory or loop [9]. They are essential for electric circuit design. There is no apparent quantum analog as quantum circuits do not form loops, despite some explorations [80]. Specifically, an output from a quantum process cannot be an input again unless it is trivial, i.e., it is a fixed point of the process. This relates to the quantum closed timeline curve [81]. However, using Bell states and Bell measurements, loops can be formed [17]; as we have seen, a Bell state or ebit is expressed as half of a loop. The difficult aspect is that there are Pauli byproducts in Bell measurements. In addition, using measurements makes the process non-unitary. At present, it is unclear what could be the proper quantum notion of loop, leading to a quantum analog of classical sequential circuits.

## Figures and Tables

**Table 1:** Comparison of three basic types of constructions of QPU. The type-III is used in QvN.

	Qubit	Gate
type-I	time	space
type-II	space	time
type-III	spacetime	spacetime

**Table 2:** Comparison between QvN and MBQC.

	Byproduct	Type	Quantum Program	Switchability
TBQC	Clifford	2-bit	qubit gates	no
MBQC	Pauli	1-bit	CZ	no
QvN	Pauli	covariant	SU(d)	yes

**Table 3:** Comparison between DQC and QvN.

	User/Verifier	Host/Prover
DQC	[U]	|U〉
QvN	|U〉	|U〉, [U]

## Data Availability

No new data were created or analyzed in this study. Data sharing is not applicable to this article.
